# Identification of superior reference genes for data normalisation of expression studies via quantitative PCR in hybrid roses (*Rosa hybrida*)

**DOI:** 10.1186/1756-0500-4-518

**Published:** 2011-11-28

**Authors:** Maik Klie, Thomas Debener

**Affiliations:** 1 Department of Molecular Plant Breeding, Institute for Plant Genetics, Leibniz Universität Hannover, Herrenhäuser Str. 2, 30419 Hannover, Germany

## Abstract

**Background:**

Gene expression studies are a prerequisite for understanding the biological function of genes. Because of its high sensitivity and easy use, quantitative PCR (qPCR) has become the gold standard for gene expression quantification. To normalise qPCR measurements between samples, the most prominent technique is the use of stably expressed endogenous control genes, the so called reference genes. However, recent studies show there is no universal reference gene for all biological questions. Roses are important ornamental plants for which there has been no evaluation of useful reference genes for gene expression studies.

**Results:**

We used three different algorithms (BestKeeper, geNorm and NormFinder) to validate the expression stability of nine candidate reference genes in different rose tissues from three different genotypes of *Rosa hybrida *and in leaves treated with various stress factors. The candidate genes comprised the classical "housekeeping genes" (*Actin, EF-1α, GAPDH*, *Tubulin *and *Ubiquitin*), and genes showing stable expression in studies in *Arabidopsis *(*PP2A, SAND, TIP *and *UBC*). The programs identified no single gene that showed stable expression under all of the conditions tested, and the individual rankings of the genes differed between the algorithms. Nevertheless the new candidate genes, specifically, *PP2A *and *UBC*, were ranked higher as compared to the other traditional reference genes. In general, *Tubulin *showed the most variable expression and should be avoided as a reference gene.

**Conclusions:**

Reference genes evaluated as suitable in experiments with *Arabidopsis thaliana *were stably expressed in roses under various experimental conditions. In most cases, these genes outperformed conventional reference genes, such as *EF1-α *and *Tubulin*. We identified *PP2A*, *SAND *and *UBC *as suitable reference genes, which in different combinations may be used for normalisation in expression analyses via qPCR for different rose tissues and stress treatments. However, the vast genetic variation found within the genus *Rosa*, including differences in ploidy levels, might also influence expression stability of reference genes, so that future research should also consider different genotypes and ploidy levels.

## Background

Roses are one of the economically most important ornamentals worldwide. They are produced as cut and potted plants and garden and landscaping plants with a production value of 24 billion Euros from 1995 to 2007 [1]. Other, less prominent uses include medicinal applications or the consumption in teas and soups [2]. Apart from the beauty of their flowers, roses are also admired for their delicate scent. Their scent is composed of a number of metabolites, which are partly derived from genes unique to roses [3]. Other characteristics important for rose breeding and cultivation are tolerance or resistance to abiotic stress (e.g., cold and heat) and diseases such as black spot, powdery mildew and downy mildew [4]. Genetic and molecular analyses led to the identification of candidate genes for some of these characteristics [3,5]. To link the phenotypic information of these characteristics to the function of the candidate genes, gene expression studies have provided crucial information on gene function. The quantification of gene expression via quantitative PCR (qPCR) is the most prominent technique to date [6].

Quantitative PCR has become the gold standard for the quantitative analysis of nucleic acids because of its high sensitivity, adequate reproducibility, broad quantification range and ease of use. Nevertheless, there are still numerous sources of experimental error, including tissue sampling or RNA integrity. A lack of experimental standardization will directly affect the reproducibility and integrity of biological replications of qPCR experiments. To facilitate the standardisation of qPCR, the MIQE (minimum information for publication of quantitative real-time PCR experiments) guidelines were proposed [7]. These guidelines provided a framework for how to conduct RT-qPCR experiments to reduce experimental errors.

A major problem of qPCR is data normalisation which strongly influences the variability among repeat experiments and therefore determines the reliability of gene expression differences between samples. Data normalisation is usually established by including stably expressed reference genes to correct an assay for sample-to-sample variation in reaction efficiency and sample quantity. The perfect reference gene should display stable expression in all tissues and in all developmental and physiological conditions of an organism [8]. The most popular reference genes are the so-called "housekeeping genes", such as *Actin *or *GAPDH*, which have been used as controls for northern-blotting or semi-quantitative RT-PCR. Recently, several studies have shown that many of these genes are not suitable for qPCR, as their expression might be changed by developmental and environmental factors, e.g., stress factors [9-11]. Because the effects of these factors are strongly species specific, suitable reference genes have to be experimentally identified for each target organism and each particular biological question. Suitable reference genes are only available for model plants with sequenced genomes, such as *Arabidopsis *[10] or *Medicago *[12], or important crops, such as wheat [13], barley [14] or tomato [15,16]. Nevertheless, RT-qPCR is routinely used in several research projects on numerous plant species. The majority of those studies are based on expression normalisation using reference genes that have not been properly validated for expression stability [17].

Several software tools are available to validate the expression stability of potential reference genes. Among the most widely used software programs are BestKeeper [18], geNorm [8] and NormFinder [19]. The BestKeeper algorithm performs repeated pairwise correlation and regression analysis of a given gene to all other genes. The geNorm program ranks reference genes based on a stepwise elimination of the least stable gene using the gene expression stability measure (M), where M is the average pairwise variation of an individual to other genes. Finally, NormFinder identifies the two reference genes that show the lowest inter- and intragroup variation by fitting a linear mixed-effect model.

In the present study, we have used the software tools described above to validate the expression of seven putative reference genes for *Rosa hybrida *across different tissues and stress factors. The genes were ranked within each experimental setup and for each software tool. We identified a set of reference genes or gene combinations for gene expression studies in hybrid roses that are superior to frequently used traditional reference genes, such as *Actin *or *GAPDH*.

## Results

### Identification of the reference gene candidates for hybrid roses

The candidate genes comprised *Actin*, *EF1-α*, *GAPDH*, *Tubulin *and *Ubiquitin*, which were frequently used as reference genes for normalisation of qPCR-data in the past [17], and additionally genes (*PP2A*, *SAND*, *TIP *and *UBC*) showing highly stable expression levels in *Arabidopsis thaliana *[10].

Homologues to these candidate genes were identified among a collection of rose leaf ESTs based on the preliminary annotations available for the EST collection (*Actin*, *EF1-a*, *GAPDH*, *Tubulin *and *Ubiquitin*) or by BLAST searches (*PP2A*, *SAND*, *TIP *and *UBC*) using *Arabidopsis *sequences (Table 1). The reference genes *PP2A*, *SAND*, *TIP *and *UBC *were selected because of their stable expression in microarray studies in *Arabidopsis thaliana *[10]. All sequences comprise the full coding sequence extending into the 5'and 3' UTRs (data not shown).

**Table 1:** The description of the rose candidate reference genes.

Gene	GenBank Accession		Primer sequence (5'-3')	Position	Amplicon	Efficiency
*EF1-α* (elongation factor 1α)	JN399225	F R	ACACCTCCCACATTGCTGTTA CTTCAAGAACTTGGGCTCCTT	1055-1075 1129-1149	95 nt	0.98

*GAPDH* (glyceraldehyde-3-phosphate dehydrogenase)	JN399220	F R	TATGACCAGATCAAGGCTGCT ACCAATGAAGTCGGTTGACAC	769-789 850-870	102 nt	0.97

*PP2A* (protein phosphatase 2A)	JN399224	F R	TGTCACTGCATCAAAGGACAG GACGAATTGTCTTCTCCACCA	1557-1577 1646-1666	110 nt	0.98

*SAND* (SAND-family protein)	JN399228	F R	GTGTTGAGGAGTTGCCTCTTG AACCTGTCGGGAGAATCTGTT	830-850 906-926	97 nt	0.98

*TIP* (TIP41-like protein)	JN399221	F R	GAATCCACGGCTGGGAAA CAGTTCGTGGGTGGAGGAGTT	77-94 121-141	65 nt	0.93

*Tubulin*	JN399223	F R	GTACATGGCCTGCTGTTTGAT ATGGTACGCTTGGTCTTGATG	900-920 969-989	90 nt	0.99

*UBC* (ubiquitin conjugating protein)	JN399227	F R	GCCAGAGATTGCCCATATGTA TCACAGAGTCCTAGCAGCACA	360-380 448-468	109 nt	0.97

The sequences were derived from a collection of 44343 ESTs generated by 454 sequencing of cDNAs from healthy and stressed rose leaves available in the lab. The primer sequences, their positions, the length of their product and their reaction efficiency in quantitative PCR (qPCR) as estimated by the LRE analyser are given.

### Various RNA samples for the validation of the reference gene candidates

RNA was extracted from the leaves, roots and immature flower buds of hybrid roses of three different genotypes (93/1-119, 94/1-30 and 94/1-97). The leaves stressed by wounding, heat shock and black spot inoculation were only harvested from genotype 93/1-119. For each treatment, tissues from three plants per genotype were separately harvested to generate three biological replicates. Each biological sample comprised two independent RNA extractions, which were pooled prior to cDNA synthesis. Each cDNA, therefore, represents one biological replicate from which six technical replicates were generated during qPCR.

### Primer efficiency of the qPCR and the expression level of the candidate reference genes

The reaction efficiency was estimated per well and for each run using the LRE (Linear Regression Efficiency)-Analyser (see Methods). The mean primer efficiency varied from 0.93 for *TIP *to 0.99 for *Tubulin *(Table 1). *Actin *and *Ubiquitin *were excluded from further analyses because their amplification efficiencies were poor, and several publications [9-11] report a significant regulation of these two genes.

**Figure 1:**
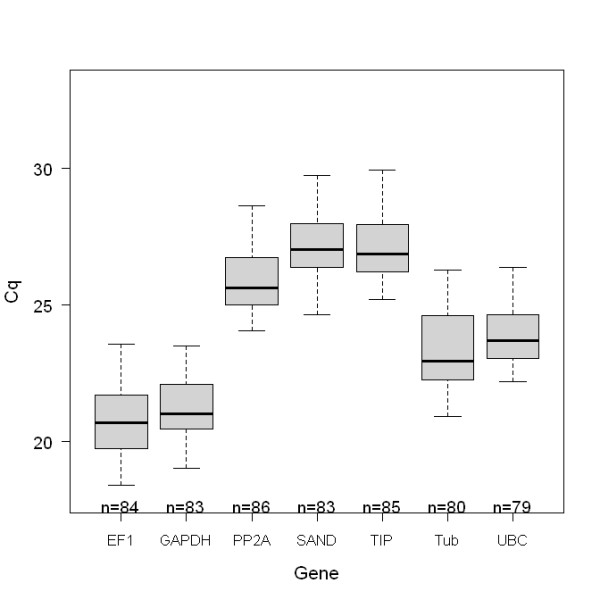
**The expression levels of the reference candidates over all treatments**. The boxplot graphs show the measured quantification cycles (Cq) for all candidate genes comprising all genotypes (93/1-119, 94/1-30 and 94/1-97), all tissues (leaves, roots and immature flower buds) and all treatments (heat shock, wounding, black spot inoculation for 2 h and 3 d) of genotype 93/1-119. The boxes represent 25 and 75 quartiles, the whisker caps indicate 10 and 90 percentiles, and the medians are indicated by the line. The number of included Cqs is indicated by n.

Based on the raw Cq values of all the candidate genes analysed for all treatments (Figure 1), different expression profiles were observed. The candidate genes *EF-1α *and *GAPDH *had the lowest Cq, indicating the highest level of expression. *Tubulin *and *UBC *were moderately expressed, whereas *PP2A*, *SAND *and *TIP *were expressed at low levels. Figure 1 also shows that the measured Cq values for *UBC *expression were the least variable among all of the reference gene candidates tested.

### The expression profile of the reference gene candidates is influenced by experimental conditions

The stress treatments slightly reduced the expression levels of the reference genes as compared with the genes in healthy, untreated leaves, as observed by an average increase of the Cq values of 2.0 for wounding (*p *= 0.001), 2.7 for heat stress (*p *= 0.001), 1.6 for a 2 h black spot inoculation (*p *= 0.008), and 1.4 for a 3-day black spot inoculation (*p *= 0.02).

### Expression stability of the reference gene candidates among different rose tissues and under several stress conditions

For validation of the candidate gene expression stability, the qPCR-data, which were measured for each biological sample with six technical replicates (Table 2), were analysed using the BestKeeper, geNorm and NormFinder software.

**Table 2:** Summary of the experimental conditions comprising the genotypes, tissues, treatments and the number of biological and technical replicates used in the present study.

Genotype	Tissue/Treatment	No. of biological replicate	No. of technical replicates per biological replicate	Whole datasets	Different tissues	Stressed leaves
94/1-30	leave	3	6	+	+	-
	floral bud	3	6	+	+	-
	root	3	6	+	+	-

94/1-97	leave	3	6	+	+	-
	floral bud	3	6	+	+	-
	root	3	6	+	+	-

93/1-119	leave	3	6	+	+	+
	floral bud	3	6	+	+	-
	root	3	6	+	+	-
	wounded leave	3	6	+	-	+
	heat stressed leave	3	6	+	-	+
	leave inoculated with black spot	3	6	+	-	+

For each tissue or treatment three biological replicates were tested with six technical replicates. The technical replicates comprised of three independent qPCR runs in which each amplification reaction was repeated once, respectively. The pluses (+) sum up the samples of the corresponding datasets of the stability analysis.

For the analyses by BestKeeper, the Cq values and primer efficiencies were used. As BestKeeper is designed to determine a reliable normalisation factor but not the independent suitability of each reference gene, we ranked the genes according to their standard deviation, coefficient of variance and coefficient of determination according to the BestKeeper index (Table 3). The most stable gene in both the whole dataset and in the separate comparisons across the analysed tissues was *UBC*. *UBC *was also ranked at position two for stressed leaves, where *GAPDH *performed best. Considering the whole dataset and data from stressed leaves, *Tubulin *was the least stable gene, whereas *TIP *was ranked last in the dataset comparing different tissues (Table 3).

**Table 3:** Ranking of the reference gene candidates by BestKeeper, geNorm, NormFinder and RankAggreg.

Rank position	Whole dataset				Different tissues				Stressed leaves			
						
	Best-Keeper	geNorm (M)	NormFinder (Stab. val.)	Rank-Aggreg	Best-Keeper	geNorm (M)	NormFinder (Stab. val.)	Rank-Aggreg	Best-Keeper	geNorm (M)	NormFinder (Stab. val.)	Rank-Aggreg
1	*UBC*	*UBC* 0.54	*PP2A* 0.19	*UBC*	*UBC*	*PP2A* 0.48	*PP2A* 0.14	*PP2A*	*GAPDH*	*SAND* 0.44	*PP2A* 0.11	*SAND*
2	*GAPDH*	*PP2A* 0.55	*UBC* 0.21	*PP2A*	*PP2A*	*UBC* 0.51	*UBC* 0.18	*UBC*	*UBC*	*TIP* 0.45	*UBC* 0.12	*UBC*
3	*PP2A*	*SAND* 0.6	*SAND* 0.27	*SAND*	*SAND*	*GAPDH* 0.56	*GAPDH* 0.24	*GAPDH*	*TIP*	*UBC* 0.47	*SAND* 0.12	*TIP*
4	*TIP*	*TIP* 0.62	*EF1-α* 0.31	*TIP*	*Tubulin*	*Tubulin* 0.59	*TIP* 0.27	*Tubulin*	*PP2A*	*EF1-α* 0.49	*TIP* 0.19	*PP2A*
5	*SAND*	*EF1-α* 0.66	*GAPDH* 0.33	*GAPDH*	*GAPDH*	*TIP* 0.59	*Tubulin* 0.28	*TIP*	*SAND*	*PP2A* 0.51	*EF1-α* 0.24	*EF1-α*
6	*EF1-α*	*GAPDH* 0.68	*TIP* 0.33	*EF1-*α	*EF1-α*	*SAND* 0.61	*SAND* 0.3	*SAND*	*EF1-α*	*GAPDH* 0.66	*Tubulin* 0.33	*GAPDH*
7	*Tubulin*	*Tubulin* 0.81	*Tubulin* 0.4	*Tubulin*	*TIP*	*EF1-α* 0.66	*EF1-α* 0.33	*EF1-*α	*Tubulin*	*Tubulin* 0.67	*GAPDH* 0.37	*Tubulin*
Best comb.		*SAND/UBC* 0.39	*EF1-α/UBC* 0.15			*SAND/TIP* 0.37	*TIP/Tubulin* 0.08			*SAND/TIP* 0.27	*SAND/UBC* 0.07	

The analyses were conducted for either the whole dataset, different rose tissues (leaves, flower buds and roots) or for stressed leaves. The best comb. is the proposed best combination of two genes given by the respective algorithm.

For the analysis of the candidate genes with geNorm and NormFinder, the Cq values were transformed into relative quantities (RQ). The geNorm algorithm estimates the average pairwise variation for one gene with all other genes of a given group of samples as a measure of gene expression stability (M); therefore, the lower the value, the more stable the gene is expressed. Because the highly variable expression of one particular gene influences the M-value of all other genes, geNorm performs a stepwise exclusion of the least stably expressed gene. In this analysis, the best performing reference gene for the whole dataset was *UBC*, whereas *PP2A *has the lowest M value for the dataset comparing different tissues (Table 3). For the dataset comparing the stressed leaves to the untreated leaves, the most stable gene was *SAND*. The least stable gene for the whole dataset and for stressed leaves was *Tubulin*. For the comparison of different tissues, the least stable gene was *EF1-α*. The results for the best combination of genes were different from those of the best two single genes for each dataset. In addition, the geNorm software proposes a suitable number of reference gene combinations by estimating the pairwise variation between two normalisation factors and considering an increasing number of genes. The authors do not recommend the addition of further reference genes for values below a cut-off value of 0.15. For the whole dataset, two reference genes provided a sufficient degree of normalisation, as exemplified by the combination of *SAND *and *UBC *with a value of 0.114.

The RQs were log transformed for the analyses by NormFinder. The NormFinder algorithm uses statistical linear mixed effects modelling to identify those genes with the smallest intra- and intergroup variation. The most stable gene for each dataset was *PP2A*, whereas the least stable genes were *Tubulin *for the whole dataset, *EF1-α *for the comparison of different tissues and *GAPDH *for stressed leaves (Table 3). The best combination of two reference genes did not comprise the best two single genes for each dataset because of the high intergroup variation.

As the individual ranking of the three algorithms differ to each other a rank aggregation by RankAggreg was used to connect the results of each list with each other (Table 3). If we apply this method to the comparison between different rose tissues, we get the following ideal order: *PP2A*, *UBC*, *GAPDH*, *Tubulin*, *TIP*, *SAND *and *EF1-α*. However, for the comparison of stressed to healthy leaves, the order was *SAND*, *UBC*, *TIP*, *PP2A*, *EF1-α*, *GAPDH *and *Tubulin*.

## Discussion

The analysis of gene expression in different tissues, developmental stages and environmental conditions is a major aspect of the functional analysis of genes. The most commonly used and, currently, the most accurate technology for gene expression analysis is qPCR, which is a method that combines high specificity and extremely high sensitivity [6]. However, the high sensitivity of this method might lead to experimental errors. The factors that strongly influence the variability of qPCR experiments are biological materials sampling, RNA extraction and cDNA synthesis methods, PCR primer design and PCR conditions, the efficiency of the qPCR reactions and normalisation of the qPCR data [7]. For normalisation, the expression levels of the reference genes, which have constant expression across all samples, are analysed along with the genes of interest. However, detailed analyses of various commonly used reference genes, such as *Actin*, have revealed that many of these genes are significantly influenced by experimental conditions [20-23]. Therefore, many authors agree that the reference genes need to be validated for each plant species and for each specific experimental setup [24].

Until recently, there have only been a few publications available that use the RT-qPCR technique for gene expression analyses in roses [25,26]. None of the studies have verified the applicability of their reference genes for gene expression analysis of different rose tissues prior to normalisation. As the reliability of those experiments depends on normalisation, the suboptimal performance of the reference genes has to be considered as a major source of error in the entire qPCR assay [17].

Here, we describe the analysis of seven putative reference genes to improve the relative quantification by qPCR for the gene expression analysis in roses. Using three algorithms (BestKeeper, geNorm and NormFinder), we evaluated the expression stability of the remaining seven genes (*GAPDH*, *PP2A*, *SAND*, *TIP*, *UBC*, *Ubiquitin *and *Tubulin*) in different rose tissues from three individual genotypes, including young leaves, roots and immature flower buds and the leaves of genotype 93/1-119 subjected to various stresses, such as heat, wounding and inoculation with the conidia of black spot.

In the analysis of our datasets, we found that no reference gene had an optimal performance across all of the conditions tested. The NormFinder program ranked *PP2A *as the best reference gene when we analysed the whole dataset, combining all stress treatments and tissues. In contrast, other genes were evaluated to be more stable under stress conditions, such as *SAND *and *TIP*, by the geNorm software or *GAPDH *and *UBC *by the BestKeeper program. Still, another picture emerges when the expression of candidate genes in different rose tissues were compared. However, all three programs ranked *PP2A *and *UBC *as the two best single reference genes.

There are two probable explanations for this observation. The first explanation is that there are no universal reference genes for every biological experiment analysed. This opinion has been expressed in various publications where differences in the performance of reference genes were found in different tissues, at different developmental stages and under different environmental conditions [8-10]. In our study, the stress treatments (heat, wounding and pathogen inoculation) significantly reduced the expression level of all seven tested candidate genes as compared with healthy untreated leaves. However, the magnitude of the reduction differed from gene to gene and from stress treatment to stress treatment. Notably, the expression of a gene might even be changed due to genetic variability among the plants analysed [27].

The other explanation is that there are differences in the algorithms used by the three computer programs. It has been reported several times that different algorithms applied to the same datasets produce different outputs with respect to the most suitable reference gene or reference gene combinations [27,28]. As most publications report the analyses of gene numbers similar to ours a higher number of reference genes could eventually lead to a stronger overlap between the algorithms.

The possible solutions to this dilemma would either be to restrict the search for optimal reference genes to a single software tool or a compromise based on the combination of the ranks made by several programs by rank aggregation [29]. If we apply this solution to the comparison of different rose tissues and stress treatments from this study, we get three different "optimal ordering lists". In each list UBC is present among the best three single genes. Nevertheless the other two genes differ.

For data normalisation via internal control genes, the use of at least three validated reference genes is recommended by [8]. An optimal experiment would include all genes of interest and the appropriate reference genes in the same PCR reaction plate [30]. A disadvantage of such an experimental setup is the limitation of the number of genes that can be analysed simultaneously. The geNorm algorithm suggests an optimal reference gene number for data normalisation. Based on our results, two validated reference genes are sufficient for accurate data normalisation.

Therefore, for the rose, we propose the use of *PP2A*, *UBC *(and *GAPDH*) for the expression analysis of different tissues and *SAND*, *UBC *(and *TIP*) for the analysis of stress treatments such as wounding, heat stress and infection by pathogens.

## Conclusions

We have identified *PP2A*, *UBC *and *SAND *as suitable reference genes that may be used in different combinations for the normalisation of expression analyses via qPCR for different rose tissues and stress treatments. According to [10], these genes belong to a new generation of reference genes and outperform the conventional reference genes (*EF1*-α and *Tubulin*) found in *Arabidopsis thaliana*. Therefore, the use of the available expression data from the microarray studies of model plants, such as *Arabidopsis*, is a helpful tool to identify new reference gene candidates, even in distant plant families, until comparable data for roses become available. Nevertheless, under certain conditions, the traditional reference genes might also be appropriate candidates, as shown by [27] and [28].

Although we used three different genotypes for our analyses we are aware that the vast genetic variation found within the genus *Rosa*, including differences in ploidy levels, might also influence expression stability of reference genes. This might be an interesting question for future research.

## Methods

### Plant material

The three different diploid genotypes of *Rosa hybrida *(93/1-119, 94/1-30 and 94/1-97) described by [31], were cultivated in 12 cm pots in a greenhouse under a 16 h light/8 h dark cycle, with a constant temperature of 22°C. Young healthy leaves and flower buds and roots were harvested from three separate clones of each genotype. The roots were induced on stem cuttings in hydroculture according to [32].

The leaflets of three clones of genotype 93/1-119 were placed in plastic boxes on water-saturated tissue paper. The stress treatment was conducted on the third to fifth unfolded leaves. For wounding, the leaves were cut with a scalpel and stored for 2 h at 20°C. Heat stress was applied by placing the wet plastic boxes at 42°C for 1 h with subsequent harvesting of tissues. Pathogen stress was applied by the inoculation of leaflets with the conidia of *Diplocarpon rosae *(black spot) race DortE4 at a density of 2 × 10^5 ^conidia/ml [33] and incubation for 2 h and 3 d at 20°C, respectively.

All samples were immediately frozen in liquid nitrogen after harvest and stored at -80°C for less than one month prior to the RNA extraction.

### Total RNA extraction and cDNA synthesis

The tissues were frozen in liquid nitrogen and ground for 1.5 min using a bead mill at 26 s^-1^. Total RNA was extracted from 30 mg of tissue in RP buffer using the InviTrap^® ^Spin Plant RNA Mini Kit (Invitek GmbH, Berlin) according to the manufacturer's instructions. The remaining DNA was removed by DNase treatment using the DNA-*free™ *Kit (Ambion Inc., Austin).

The RNA concentration was assessed spectrophotometrically at 260 nm and was checked for purity by determining the OD 260 nm/280 nm and the OD 260 nm/230 nm ratios, respectively. The RNA quality was assessed by gel electrophoresis and for a subset of samples using a Bioanalyser 2100 lab chip (Agilent, Santa Clara).

For each sample, 300 ng of total RNA was reverse-transcribed using the High-Capacity cDNA Reverse Transcription Kit (Applied Biosystems, Austin) and 1 μg of an oligo-(dT)_18 _Primer (Fermentas, St. Leon-Rot) according to the manufacturer's instructions. The cDNAs were diluted 1:30 with nuclease-free water prior to the qPCR analyses.

### Selection of rose sequences

The potential homologues to the nine published reference genes in the rose were identified among a collection of 44343 ESTs generated by 454 cDNA sequences from healthy and stressed rose leaves (Debener unpublished).

The candidate genes comprised the classical reference genes *Actin*, *EF1-α *(elongation factor 1 α), *GAPDH *(glyceraldehyde-3-phosphate dehydrogenase), *Tubulin *and *Ubiquitin *and genes, such as *PP2A *(protein phosphatase 2A), *SAND *(SAND-family protein), *TIP *(TIP41-like protein) and *UBC *(ubiquitin conjugating protein), that were previously shown to have highly stable expression levels by microarray analyses in *Arabidopsis thaliana *[10].

Open reading frames and 5'and 3' UTR regions were predicted using the FGENESH algorithm [34] with specifications for dicot plants.

### PCR primer design

The primers (Table 1) for qPCR were designed to match sequences close to the predicted 3'-UTR using Primer3 software [35] with the following specifications: optimal TM at 60°C, optimal primer length of 21 nt, optimal amplicon length of 100 bp, GC content between 45% and 55% and a ΔG of -9 kcal/mol. The primers were analysed for dimers using PerlPrimer software [36]. The primer specificity was determined by BLAST searches of the entire collection of rose ESTs available in the lab. The performance of the primers was tested in PCR reactions with both gDNA and cDNA as substrates.

### Quantitative PCR and data analyses

Each biological sample was examined in six technical replicates (Table 2), which comprised of three independent PCR runs and in each run every amplification reaction was repeated once.

The amplification reactions were performed on transparent 0.1 ml 96-well plates (Applied Biosystems, Austin) using SYBR Green detection chemistry and run on the StepOnePlus™ System (Applied Biosystems, Austin). The reactions were prepared in a total volume of 10 μl containing 2 μl of template, 1 μl of each amplification primer [0.25 nM], 5 μl of 2X MESA Fast SYBR MasterMix (Eurogentec, Cologne) and 1 μl of nuclease-free water. The water-only controls included 3 μl of nuclease-free water instead of a cDNA template and were run for each primer pair on each plate. The cycling conditions were set as follows: initial denaturation for 5 min at 95°C, followed by 45 cycles of 15 s at 95°C and 45 s at 60°C, respectively. The amplification specificity for each primer pair was tested by a melting curve analysis ranging from 60 to 90°C with temperature steps of 0.5°C (Additional file 1). The PCR products were analysed on 3% agarose gels. StepOne™ Software was used to perform the baseline correction, and a common threshold for determining the quantification cycle (Cq) was set to 0.5. The data were exported to MS Excel for further statistical analysis. The efficiency of each primer pair was estimated for each reaction using the LRE-Analyser [37].

To test for a negative regulatory effect of stress treatments on the potential reference genes, a one-sided Dunnett-procedure included in the R [38] software package multcomp was used. Therefore, the Cq values for the different stress factors (wounding, heat stress, inoculation for 2 h and 3 d) for all genes were compared to the Cq values for these genes in the untreated leaves using a significance level of 95%.

The suitability of the candidate reference genes was evaluated by three statistical approaches. The BestKeeper algorithm uses descriptive statistics to estimate a reference gene index. Therefore, only Cq values were needed. For the other two (geNorm and NormFinder), the Cqs were transformed to relative quantities (RQ) using the following formula according to [39]:

$$RQ={\bar{E}}_{g}^{\left(Cq_{\min g}-Cq_{gi}\right)}$$

RQ is the relative quantity of the gene of interest (*g*), $\bar{E}$ is the mean PCR efficiency, *min *is the lowest estimated Cq of a gene (*g*) under all treatments and *i *is the specific sample. The resulting RQs were imported to the programs mentioned above and analysed according to the developer's instructions. The ranking of the three different programs was used to generate an ideal order applying the RankAggreg algorithm by [29].

## Competing interests

The authors declare that they have no competing interests.

## Authors' contributions

MK did the experimental work and participated in the writing of the manuscript. TD planned and designed the project and participated in the writing of the manuscript. Both authors have read and approved the final manuscript.

## Supplementary Material

Additional file 1 **Melting curves of the tested reference genes amplified with the primer pairs listed in Table **1. Click here for file
