# Supplementary material for: Identification of superior reference genes for data normalisation of expression studies via quantitative PCR in hybrid roses (Rosa hybrida)
Source: BMC Res Notes. 2011 Nov 28;4:518. doi: 10.1186/1756-0500-4-518 (PMC3248381; doi:10.1186/1756-0500-4-518)

Additional file 1 – Melting curves of the tested reference genes amplified with the primer pairs listed in Table 1.

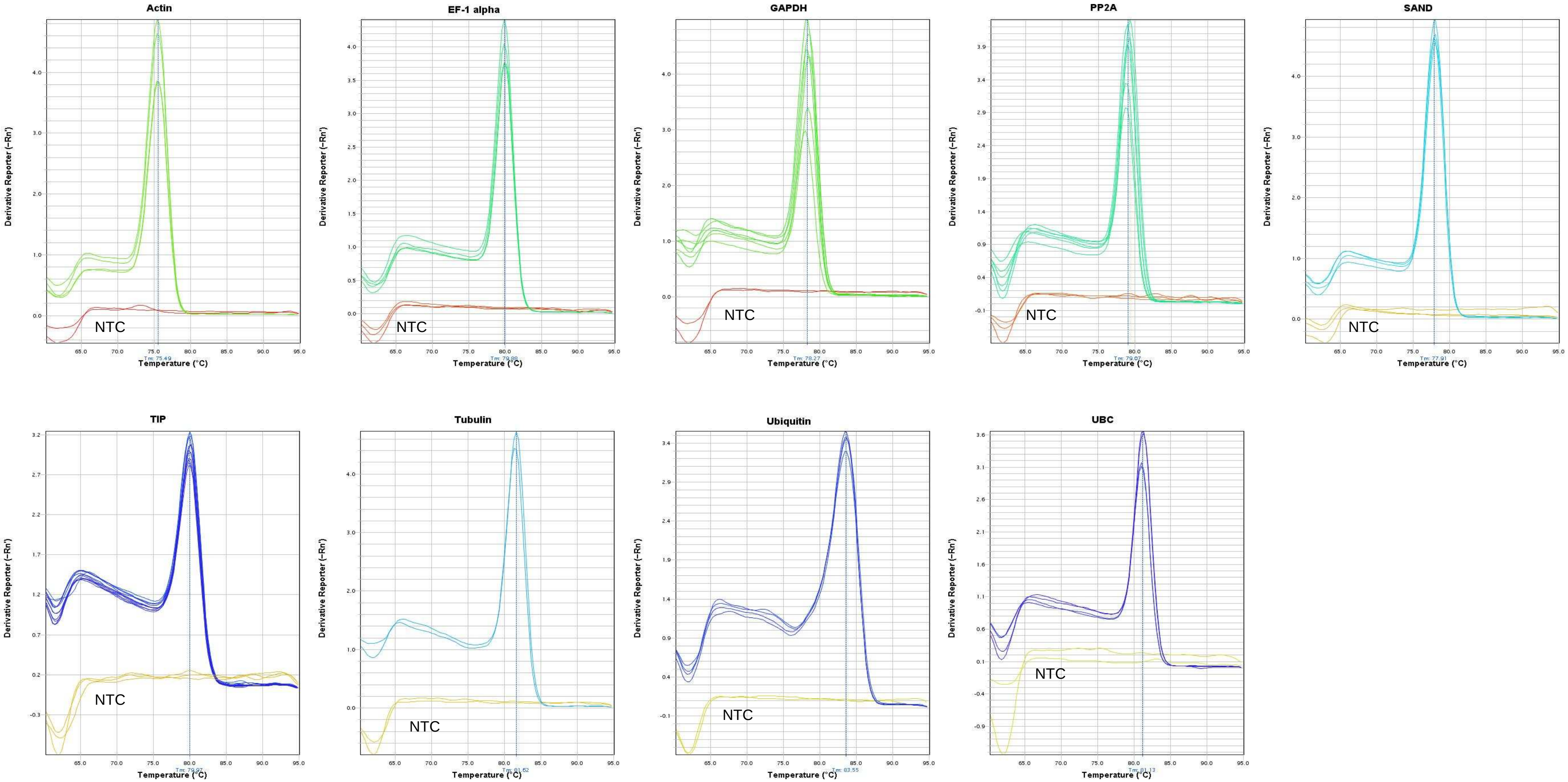

Supplement: Additional file 1 — Melting curves of the tested reference genes amplified with the primer pairs listed in Table 1. [file 1756-0500-4-518-S1.PDF]
